# Overexpression of *Stanniocalcin 2* Protects Differentiated QM7 Cells from H_2_O_2_-Induced Oxidative Damage

**DOI:** 10.3390/ijms27125471

**Published:** 2026-06-17

**Authors:** Sarang Choi, Sangsu Shin

**Affiliations:** 1 Department of Animal Science and Biotechnology, Kyungpook National University, Sangju 37224, Republic of Korea; lagc94@knu.ac.kr; 2 Research Institute for Innovative Animal Science, Kyungpook National University, Sangju 37224, Republic of Korea

**Keywords:** *Stanniocalcin 2*, oxidative stress, QM7 cell line, skeletal muscle, myotube, differentiation

## Abstract

Oxidative stress, caused by excessive reactive oxygen species (ROS) accumulation, is a major factor in muscle cell damage and muscle atrophy-related disorders. Although *Stanniocalcin 2* (*STC2*) is involved in cellular stress and exhibits cytoprotective effects in various cell types, its role in skeletal muscle cells during oxidative stress is unclear. This study investigated the effects of *STC2* overexpression in quail muscle (QM7) cells exposed to H_2_O_2_-induced oxidative stress. *STC2* expression was upregulated in non-transfected QM7 cells following H_2_O_2_ treatment. Stable *STC2*-overexpressing cells were differentiated for 4 days, and then assessed for cell viability, ROS accumulation, cell death, and myotube morphology following H_2_O_2_ treatment. Compared with control cells, *STC2*-overexpressing cells exhibited higher cell viability, reduced ROS accumulation, and decreased cell death. *STC2* overexpression also attenuated the H_2_O_2_-induced reduction in MyHC protein expression. Antioxidant-related genes, including *Superoxide Dismutase 1*, *Glutathione Peroxidase 1*, *Heme Oxygenase 1*, and *NAD(P)H Quinone Dehydrogenase 1*, were significantly upregulated in *STC2*-overexpressing cells. Compared with the control cells, nuclear factor erythroid 2-related factor 2 protein levels were not increased in *STC2*-overexpressing cells under oxidative stress conditions. These findings suggest that *STC2* overexpression alleviates oxidative stress-induced cellular damage and may contribute to protective antioxidant responses in muscle cells.

## 1. Introduction

Oxidative stress, which arises from an imbalance between the generation of reactive oxygen species (ROS) and cellular antioxidant defense mechanisms, plays a key role in both physiological and pathological processes [1]. ROS, including hydrogen peroxide (H_2_O_2_), superoxide anion (O_2_^•−^), and hydroxyl radicals (^•^OH), are byproducts of normal cellular metabolism [2], and their physiological levels are indispensable for cellular signaling and homeostasis. However, excessive ROS production induces protein, lipid, and DNA damage, causing cellular dysfunction and death [3,4]. Indeed, oxidative stress is involved in the progression of neurodegenerative disorders, cardiovascular diseases, metabolic diseases, and cancer [5,6,7,8].

Skeletal muscles, which are major organs in whole-body energy metabolism, frequently generate ROS because of their high metabolic rate and mitochondrial activity [9,10]. Physiological stimuli, e.g., exercise, can induce muscle adaptation and regeneration through transient increases in ROS levels [11]. However, excessive ROS accumulation leads to muscle protein degradation, mitochondrial damage, muscle atrophy, and reduced muscle regenerative capacity [12,13]. Moreover, in aging or muscle atrophy-related diseases like sarcopenia, cachexia, and muscular dystrophy, oxidative stress is a major cause of muscle damage and dysfunction [14,15]. Therefore, mechanisms that protect muscle cells from oxidative stress are important for muscle health and disease prevention.

Various protective mechanisms are activated in response to cellular stress, including the regulation of stress-response gene expression. The STC2 was originally identified as a hormone involved in calcium and phosphate metabolism [16,17]. Previous studies indicate that its expression increases under endoplasmic reticulum stress, H_2_O_2_-induced oxidative stress, and hypoxia, during which it inhibits stress-induced apoptosis and promotes the survival of various cell types and tissues, e.g., hepatocytes, pancreatic cells, and mesenchymal stem cells [18,19,20,21]. *STC2* also contributes to metabolic homeostasis through the regulation of glucose and lipid metabolism and is associated with metabolic disorders, such as liver disease, hypertriglyceridemia, and glycemic dysregulation [20,22,23].

However, the potential role of *STC2* in oxidative stress responses and antioxidant protection in muscle cells has not been fully elucidated, although our previous study demonstrated that *STC2* overexpression promoted myotube elongation during muscle differentiation, suggesting a functional role of *STC2* in muscle cells [24]. In addition, the established roles in the regulation of stress response and metabolism suggest that *STC2* may have important physiological functions in skeletal muscle. A previous study has also reported that *STC2* expression increases during overload-induced muscle hypertrophy [25]. Because overload-induced hypertrophy is associated with hypoxia and oxidative stress [26], *STC2* upregulation may contribute to protective responses in muscle cells under stress conditions.

This study aimed to investigate how *STC2* contributes to the viability of myogenic cells and protects against oxidative stress-induced damage. By examining the effect of *STC2* overexpression on ROS accumulation, antioxidant-related gene expression, and muscle cell death, this study suggests that *STC2* may help protect muscle cells against oxidative stress-induced cellular damage.

## 2. Results

### 2.1. Determination of Oxidative Stress Conditions and STC2 Expression in QM7 Cells

**Figure 1:**
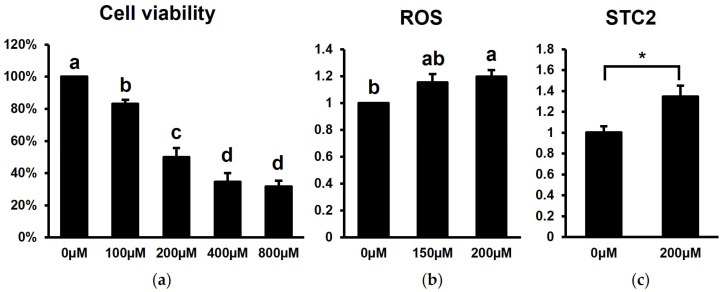
Cell viability, ROS accumulation and *STC2* expression in muscle cells after treatment with H_2_O_2_. (**a**) Cell viability was measured after treatment with H_2_O_2_ at 0-800 μM for 3 h on day 4 of differentiation. Data were analyzed by one-way ANOVA followed by Duncan’s multiple range test. (**b**) Intracellular ROS levels were determined using the DCF-DA fluorescent probe in QM7 cells on day 4 of differentiation. Data were analyzed by one-way ANOVA followed by Duncan’s multiple range test. Columns labeled with different letters indicate significant differences (*p* < 0.05). (**c**) Relative *STC2* mRNA levels in QM7 cells treated with 0 or 200 μM H_2_O_2_ for 3 h on day 4 of differentiation. Data were analyzed by Student’s *t*-test. The * indicates *p* < 0.05. n = 3 biological replicates.

To establish the conditions for H_2_O_2_-induced cytotoxicity, QM7 cells were treated with various concentrations of H_2_O_2_ on day 4 of differentiation, followed by cell viability analysis (Figure 1a). Cell viability decreased dose-dependently (*p* < 0.001) and was reduced by approximately 50% at 200 μM without causing excessive cell death in myogenic cells. Therefore, 200 μM was selected as the optimal concentration in this study. To determine whether H_2_O_2_ affected intracellular ROS levels, ROS levels were measured using DCF-DA on day 4 of differentiation (Figure 1b), which revealed a dose-dependent increase in ROS levels and a significant elevation at 200 μM of H_2_O_2_ (*p* < 0.05), which is consistent with the cell viability data. To explore the potential role of *STC2* in response to oxidative stress, *STC2* expression was analyzed in QM7 cells after treatment with 200 μM H_2_O_2_ (Figure 1c). This analysis revealed significantly increased *STC2* in cells treated with 200 μM H_2_O_2_ when compared with control cells (*p* < 0.05), suggesting that *STC2* may be an important regulatory factor during oxidative stress.

### 2.2. STC2 Overexpression Alleviates Oxidative Damage in Muscle Cells

**Figure 2:**
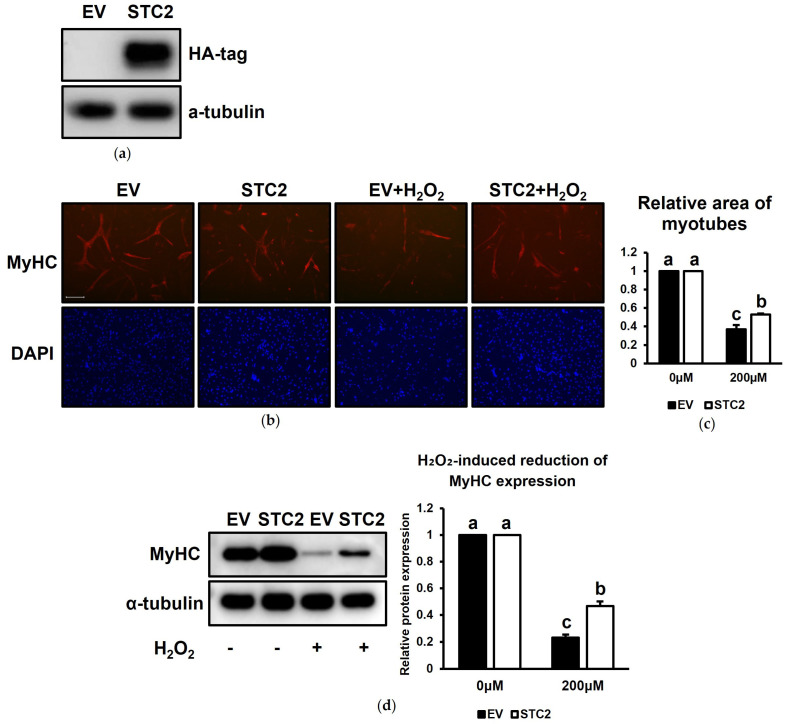
The protective effect of *STC2* overexpression in myogenic cells under H_2_O_2_-induced oxidative stress. (**a**) STC2 overexpression was confirmed using Western blotting with an anti-HA-tag antibody on day 4 of differentiation. (**b**) Immunostaining with an anti-MyHC antibody and DAPI was used to visualize myotubes (red) and nuclei (blue), respectively. (**c**) The relative myotube area (MyHC-positive) was quantified in *STC2*-overexpressing cells vs. EV controls after treatment with 200 μM H_2_O_2_ for 3 h. Scale bar: 100 μm. Columns labeled with different letters indicate significant differences (*p* < 0.05). (**d**) MyHC protein expression was analyzed by Western blotting in EV- and *STC2*-overexprssing cells with or without 200 μM H_2_O_2_ for 3 h. Columns labeled with different letters indicate significant differences (*p* < 0.05). Data were analyzed by two-way ANOVA followed by Duncan’s multiple range test. n = 3 biological replicates.

Stable *STC2*-overexpressing QM7 cells were established to investigate the role of *STC2* in H_2_O_2_-induced damage in muscle cells, and STC2 expression was confirmed using Western blot analysis on day 4 of differentiation (Figure 2a), which clearly detected the HA-tag in *STC2*-overexpressing cells when compared with control cells. To examine whether *STC2* overexpression affects myotube integrity under oxidative stress, EV- and *STC2*-overexpressing cells were differentiated, treated with or without H_2_O_2_, and stained with anti-MyHC antibodies and DAPI on day 4 (Figure 2b). While both groups showed well-developed myotubes in the absence of H_2_O_2_, *STC2*-overexpressing cells retained more myotubes after treatment with H_2_O_2_ when compared with EV cells. To quantitatively validate these observations, the area of MyHC-positive myotubes was measured (Figure 2c). Although the MyHC-positive cells were significantly reduced in both groups upon treatment with H_2_O_2_ (*p* < 0.001), the reduction was less pronounced in *STC2*-overexpressing cells when compared with the controls (*p* < 0.05). Moreover, myotube area was significantly affected by the interaction between H_2_O_2_ treatment and *STC2* overexpression (*p* < 0.05), which is consistent with Figure 2b. To further evaluate myotube integrity under oxidative stress conditions, MyHC protein levels were analyzed by Western blotting in EV- and *STC2*-overexpressing cells on day 4 of differentiation following treatment with or without H_2_O_2_ (Figure 2d). MyHC expression was significantly decreased by H_2_O_2_ treatment in both groups (*p* < 0.001); however, the reduction was less pronounced in *STC2*-overexpressing cells than in EV cells (*p* < 0.001). These findings indicate that *STC2* overexpression attenuated H_2_O_2_-induced loss of myotubes.

### 2.3. STC2-Mediated Regulation of Cell Viability, ROS Accumulation, and Cell Death

**Figure 3:**
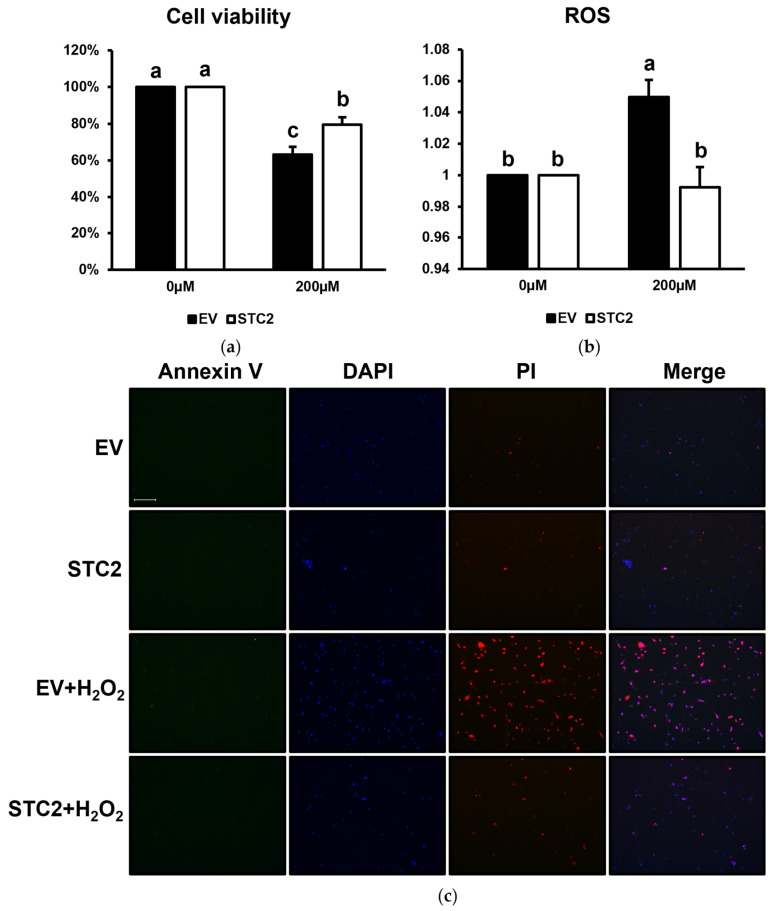
The effect of *STC2* overexpression on cell viability, ROS accumulation, and cell death in QM7 cells. (**a**) Cell viability was measured in EV and *STC2*-overexpressing cells on day 4 of differentiation. The cells were treated with 200 μM H_2_O_2_ for 3 h. (**b**) Intracellular ROS levels in EV and *STC2*-overexpressing cells were assessed using the DCF-DA fluorescent probe after treatment with 200 μM H_2_O_2_ for 3 h. (**c**) Cell death was evaluated using Annexin V (green) and propidium iodide (PI; red) staining in EV and *STC2*-overexpressing cells on day 4 of differentiation treated with 200 μM H_2_O_2_ for 3 h. Nuclei were counterstained with DAPI (blue). Scale bar: 100 μm. Data were analyzed by two-way ANOVA followed by Duncan’s multiple range test. Columns labeled with different letters indicate significant differences (*p* < 0.05). n = 3 biological replicates.

To assess whether the preservation of myotube integrity by *STC2* overexpression under oxidative stress was associated with enhanced cell survival, cell viability was analyzed following treatment with H_2_O_2_ (Figure 3a). Treatment with 200 μM H_2_O_2_ reduced the viability of EV cells to approximately 60% (*p* < 0.05). Importantly, cell viability was significantly higher in *STC2*-overexpressing cells, with a less pronounced reduction when compared with EV cells (*p* < 0.05). Next, ROS levels were measured using DCF-DA to examine whether *STC2* overexpression suppresses intracellular ROS accumulation (Figure 3b). EV cells exhibited a significant increase in ROS levels in response to increasing H_2_O_2_ concentrations, whereas this increase was attenuated in *STC2*-overexpressing cells (*p* < 0.01). The effects of H_2_O_2_ on cell viability and ROS accumulation were significantly modulated by *STC2* overexpression (*p* < 0.05 and *p* < 0.01, respectively; Figure 3a,b). Finally, Annexin V/PI analysis of whether *STC2* reduces oxidative stress-induced cell death in muscle cells revealed that upon treatment with H_2_O_2_, the EV group had an increased number of PI-positive cells, indicating late cell death or necrosis (Figure 3c). In contrast, *STC2*-overexpressing cells had a higher proportion of live cells (DAPI-positive/PI-negative) and fewer dead cells (PI-positive) when compared with EV cells. These findings indicate that *STC2* overexpression is associated with reduced ROS accumulation and reduced oxidative stress-associated cellular damage, which may contribute to the preservation of myotube integrity.

### 2.4. The Effect of STC2 Overexpression on the Expression of Oxidative Stress-Related Genes

**Figure 4:**
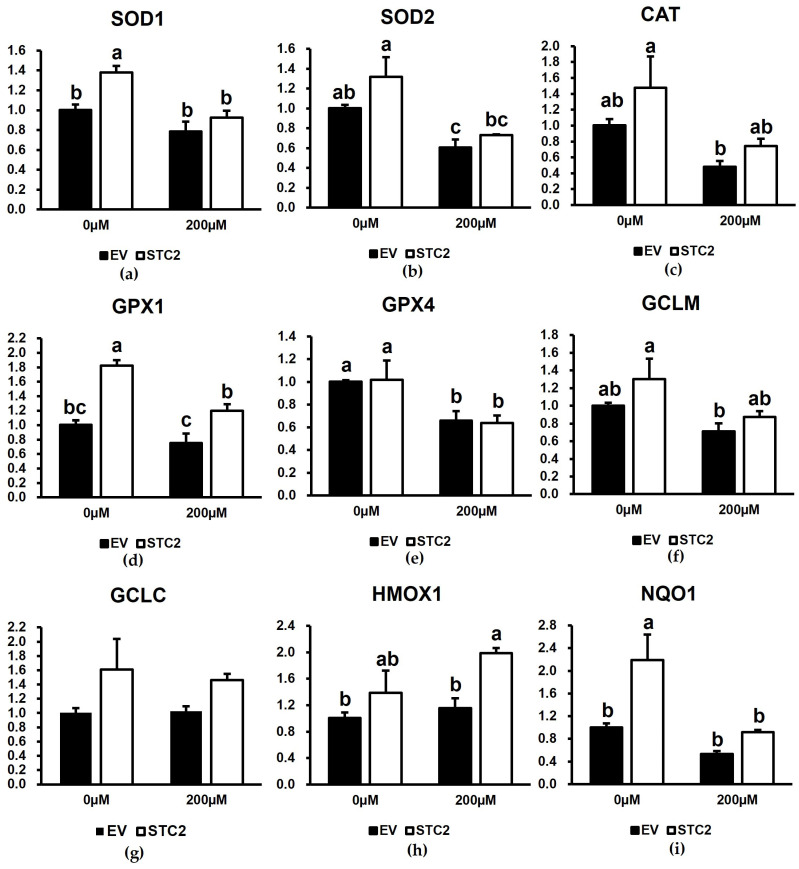
The effect of *STC2* overexpression on oxidative stress-related gene expression upon treatment with H_2_O_2_. (**a**–**i**) On day 4 of differentiation, the gene expression of *SOD1*, *SOD2*, *CAT*, *GPX1*, *GPX4*, *GCLM*, *GCLC*, *HMOX1*, and *NQO1* was analyzed in EV and *STC2*-overexpressing cells treated with 200 μM H_2_O_2_ for 3 h. Columns labeled with different letters indicate significant differences (*p* < 0.05). Columns labeled with the same letter are not significantly different. Data were analyzed by two-way ANOVA followed by Duncan’s multiple range test. n = 3 biological replicates.

To examine the effect of *STC2* overexpression on the expression of antioxidant markers, mRNA levels were analyzed in EV and *STC2*-overexpressing cells treated with or without 200 μM H_2_O_2_ (Figure 4). The expression of *Superoxide Dismutase* (*SOD*) *1*, a cytosolic antioxidant enzyme that catalyzes O_2_^•−^ conversion into H_2_O_2_ [27], was significantly increased in *STC2*-overexpressing cells when compared with EV cells (*p* < 0.01; Figure 4a). Although the expression of *SOD2*, the mitochondrial isoform of SOD [27], was not significantly different between the groups (*p* = 0.0767), it tended to be higher in *STC2*-overexpressing cells regardless of treatment with H_2_O_2_ (Figure 4b). Treatment with H_2_O_2_ downregulated both *SOD* genes in the two groups (*p* < 0.01; Figure 4a,b).

The expression of *Catalase* (*CAT*), which decomposes H_2_O_2_ into water and oxygen [28], did not differ significantly between the groups (Figure 4c). *CAT* expression decreased in both groups following treatment with H_2_O_2_ (*p* < 0.05).

The expression of *Glutathione Peroxidase (GPX) 1*, which reduces H_2_O_2_ and lipid peroxides to water [29], was significantly higher in *STC2*-overexpressing cells than EV cells (*p* < 0.001), including during treatment with H_2_O_2_ (*p* < 0.01; Figure 4d). In contrast, *GPX4*, which inhibits lipid peroxidation to prevent ferroptosis [30], was significantly reduced upon treatment with H_2_O_2_ in both groups (*p* < 0.01), and no difference was observed between the groups (Figure 4e).

*Glutamate-Cysteine Ligase Modifier Subunit* (*GCLM*) and *Glutamate-Cysteine Ligase Catalytic Subunit* (*GCLC*) constitute glutamate–cysteine ligase, the rate-limiting enzyme in glutathione biosynthesis [31]. Although *GCLM* expression was not significantly different between the groups (*p* = 0.1097), it tended to be higher in *STC2*-overexpressing cells. Treatment with H_2_O_2_ reduced *GCLM* expression in both groups (*p* < 0.05; Figure 4f). *GCLC* expression did not differ significantly between the groups (Figure 4g).

Compared with EV cells, *Heme Oxygenase 1* (*HMOX1*), a heme-degrading enzyme that generates biliverdin and contributes to antioxidant defense [32], was significantly higher in *STC2*-overexpressing cells (*p* < 0.05), with this difference persisting under treatment with H_2_O_2_ (Figure 4h). The expression of *NAD(P)H Quinone Dehydrogenase 1* (*NQO1*), which limits ROS generation by reducing quinones to hydroquinones [33], was significantly higher in *STC2*-overexpressing cells than in EV cells (*p* < 0.01; Figure 4i). Although both groups had reduced *NQO1* expression upon treatment with H_2_O_2_ (*p* < 0.01), its levels were relatively higher in *STC2*-overexpressing cells than in EV cells. Collectively, these results suggest that *STC2* overexpression is associated with increased transcript levels of antioxidant-related genes involved in superoxide detoxification, H_2_O_2_ reduction, glutathione synthesis, and quinone metabolism. These transcriptional changes may contribute to the reduced ROS accumulation observed in *STC2*-overexpressing cells under oxidative stress conditions.

### 2.5. Nuclear Factor Erythroid 2-Related Factor 2 (NRF2) Protein Expression in STC2-Overexpressing Muscle Cells

**Figure 5:**
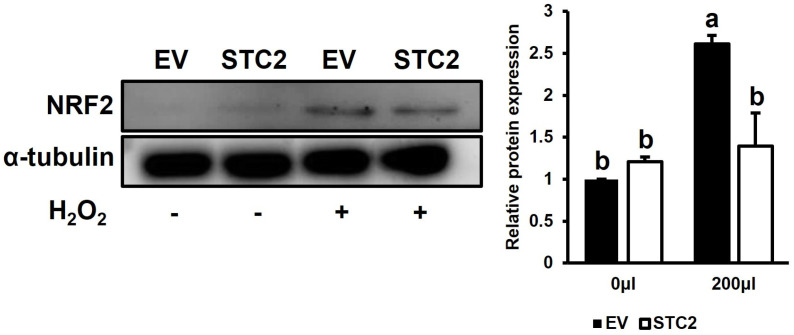
The effect of *STC2* overexpression on NRF2 expression upon treatment with H_2_O_2_. On day 4, NRF2 protein levels were analyzed using Western blotting in EV and *STC2*-overexpressing cells treated with 200 μM H_2_O_2_ for 3 h. Columns labeled with different letters indicate significant differences (*p* < 0.05). Columns labeled with the same letters are not significantly different. Data were analyzed by two-way ANOVA followed by Duncan’s multiple range test. n = 3 biological replicates.

To discover whether the upregulation of specific antioxidant-related genes was associated with NRF2 activation, NRF2 protein expression levels were analyzed by Western blotting (Figure 5). In untreated conditions, NRF2 expression was barely detectable in both groups. Treatment with H_2_O_2_ increased NRF2 protein levels in EV cells, but not in *STC2*-overexpressing cells (*p* < 0.05). Furthermore, the effect of H_2_O_2_ on NRF2 expression was dependent on *STC2* overexpression (*p* < 0.01). These results suggest that *STC2* overexpression may alter oxidative stress-induced NRF2 responses in muscle cells.

## 3. Discussion

This study investigated the protective role of *STC2* against oxidative stress in muscle cells. First, an oxidative stress model was established on day 4 of differentiation using H_2_O_2_ (Figure 1a,b). H_2_O_2_ reduced cell viability in a dose-dependent manner, confirming the induction of oxidative cytotoxicity. *STC2* expression was upregulated under oxidative stress (Figure 1c), which is consistent with previous findings in various cell types [18,21,34]. This finding suggests a potential association between *STC2* upregulation and the cellular response to oxidative stress.

This study provides evidence that *STC2* overexpression alleviates H_2_O_2_-induced cellular damage. Compared with control cells, *STC2*-overexpressing cells maintained a greater MyHC-positive myotube area, and the H_2_O_2_-induced reduction in MyHC protein expression was less pronounced in *STC2*-overexpressing cells (Figure 2). Moreover, *STC2*-overepressing cells exhibited higher viability, reduced ROS accumulation, and fewer PI-positive cells (Figure 3). Annexin V staining was not clearly detected in muscle cells, whereas PI-positive cells were prominently observed in oxidative stress conditions (Figure 3c). These findings suggest that the weak Annexin V signal may be associated with the differentiated and multinucleated characteristics of myotubes rather than simply indicating the absence of early apoptosis. During early apoptosis, Annexin V binds to phosphatidylserine (PS) on the outer leaflet of the plasma membrane [35]. PS externalization in myotubes is highly limited or transient, resulting in a weak Annexin V signal [36]. Furthermore, differentiated myotubes are multinucleated structures with complex morphology, which complicates conventional single-cell-based analysis of cell death. Quantitative assessment of Annexin V-positive cells in differentiated myotubes therefore remains technically challenging. Because of these structural limitations, the Annexin V staining results in the present study were presented as representative images rather than quantitative measurements. Accordingly, this finding primarily indicates that *STC2* overexpression reduced oxidative stress-induced cellular damage under H_2_O_2_-induced conditions. This finding is consistent with the observation that *STC2* enhances stem cell survival by inhibiting oxidative stress [21].

A key finding of this study is the upregulation of several antioxidant-related genes in *STC2*-overexpressing cells, with or without H_2_O_2_-induced stress (Figure 4). In basal conditions, the expression levels of *SOD1*, *GPX1*, and *NQO1* were higher in *STC2*-overexpressing cells (Figure 4a,d,i). Increased *SOD1* expression may facilitate superoxide conversion to H_2_O_2_, which can subsequently be reduced by increased *GPX1*, thereby promoting efficient ROS removal [37]. Moreover, increased *NQO1* expression may contribute to reduced ROS accumulation by suppressing quinone redox cycling [33]. These transcriptional changes suggest that *STC2* overexpression may support oxidative stress-induced cellular responses in muscle cells. In contrast, *STC2* overexpression did not significantly affect *GCLC* and *GCLM* expression, suggesting that *STC2* may selectively enhance the expression of enzymes related to ROS inhibition rather than glutathione synthesis. The expression levels of *GPX1* and *HMOX1* remained higher in *STC2*-overexpressing cells under oxidative stress. Previous studies have demonstrated reduced HO-1 and NQO1 expression in *STC2*-knockdown cells and that STC2 can complex with HO-1, suggesting a functional association between STC2 and HO-1-mediated antioxidant responses [38,39]. Increased *HMOX1* expression may provide additional cytoprotective effects through the generation of antioxidants, e.g., biliverdin and bilirubin during heme degradation [32]. Therefore, the increase in *HMOX1* expression observed in this study suggests that *STC2* may be involved in HO-1-related responses under oxidative stress conditions. Collectively, these findings suggest that *STC2* overexpression is associated with altered antioxidant-related gene expression and reduced ROS accumulation under oxidative stress conditions, which may contribute to the alleviation of myotube damage.

NRF2 is a key transcription factor that regulates the expression of antioxidant-related genes by binding to the promoter regions of antioxidant response element sequences [40,41]. Because many of the upregulated genes identified in this study are known NRF2 targets [42,43,44], NRF2 expression was analyzed to determine whether *STC2*-mediated cellular protection is associated with NRF2 signaling. Antioxidant-related genes were consistently upregulated under oxidative stress conditions, although NRF2 protein levels were not elevated in *STC2*-overexpressing cells. This finding suggests two possible mechanisms. First, *STC2* may function as a downstream or complementary component in NRF2 signaling in muscle cells. A recent study in hepatocellular carcinoma cells reported reduced *STC2* expression in Nrf2-deficient cells and that *STC2* overexpression enhanced NRF2 activity, suggesting that STC2 may regulate NRF2 through the Keap1-p62 signaling pathway [39]. However, NRF2 can also be activated through Keap1-independent mechanisms [45], and the precise regulatory mechanism may vary with cell type or physiological context. Second, the changes in antioxidant-related gene expression associated with *STC2* overexpression may occur independently of additional NRF2 induction. *STC2* overexpression activates PI3K/AKT and ERK1/2 signaling pathways in mesenchymal stem cells under oxidative stress, promoting cell survival [21]. Therefore, *STC2* overexpression may influence oxidative stress-associated cellular responses through mechanisms that do not require further NRF2 induction. However, further research is needed to fully understand the complex interaction between *STC2* and NRF2.

This study has several limitations. The experiments were performed using QM7 quail muscle cells. Therefore, caution is required when applying these findings to humans or other mammalian systems. In addition, the functional analyses were performed using a single *STC2*-overexpressing clone. Although the selected clone exhibited differentiation characteristics comparable to those of EV controls, potential clonal effects cannot be completely excluded. Further studies addressing potential clonal effects and using mammalian muscle cell lines, such as C2C12, are needed to validate the role of *STC2* in oxidative stress responses and antioxidant protection in muscle cells. Collectively, these findings demonstrate that *STC2* overexpression alleviates oxidative stress-induced cellular damage and supports the maintenance of differentiated muscle cells under oxidative stress conditions.

## 4. Materials and Methods

### 4.1. STC2 Vector Construction

The *STC2*-overexpression vector was constructed as described previously [25]. Briefly, the coding sequence of quail *STC2* (GenBank accession number: XM_015876362.2) was amplified using polymerase chain reaction (PCR) and cloned into a pcDNA3.1 vector using the BamHI and XbaI restriction sites. A hemagglutinin (HA) tag sequence was inserted immediately upstream of the *STC2* stop codon. The primer sets used are listed in Table 1.

### 4.2. Establishment of the Stable Cell Line

QM7 cells (American Type Culture Collection, Manassas, VA, USA) were transfected with either the *STC2*-containing vector or an empty vector (EV) using the jetOPTIMUS^®^ DNA transfection reagent (Polyplus, Illkirch-Graffenstaden, France) as per the manufacturer’s instructions. Stably transfected cells were selected with geneticin at 1 mg/mL (Santa Cruz Biotechnology, Dallas, TX, USA) for at least 2 weeks, and geneticin-resistant clones were maintained by culture in medium containing geneticin. Following geneticin selection, several independent clones were screened for *STC2* expression and myotube formation. A clone showing *STC2* expression and, differentiation characteristics comparable to EV controls was selected for subsequent experiments.

### 4.3. Cell Culture and Differentiation

Cells were cultured in Medium 199 (Gibco, Waltham, MA, USA) supplemented with 10% fetal bovine serum (Gibco), 1% chicken serum (Sigma–Aldrich, St. Louis, MO, USA), and 1% antibiotic–antimycotic (Gibco). For experiments with myotubes, cells were seeded and cultured to confluence in 6-well plates or 35 mm dishes. The medium was replaced with differentiation medium supplemented with 0.5% fetal bovine serum, 0.1% chicken serum, and 1% antibiotic–antimycotic 48 h after seeding. Cells were cultured at 37 °C, with 5% CO_2_. The differentiation medium was replaced with fresh medium on day 2. Cells were differentiated for 4 days and then used in subsequent analyses. Cells were treated with H_2_O_2_ for 3 h. The treatment duration was selected based on a previous study [46].

### 4.4. Cell Viability

On day 4 of differentiation, QM7 cells were treated with H_2_O_2_ (Samchun Chemical, Seoul, Republic of Korea) at various concentrations (0–800 μM) for 3 h, followed by two washes with phosphate-buffered saline (PBS). The cells were then incubated with 100 μL of EZ-Cytox (DoGenBio, Seoul, Republic of Korea) for 2 h, followed by absorbance reading on a GloMax Discover Multi-Microplate Reader at 450 nm (Promega Corporation, Madison, WI, USA), and the background absorbance read at 600 nm was subtracted from the absorbance reading at 450 nm.

### 4.5. Analysis of ROS Accumulation

On day 4 of differentiation, cells were treated with H_2_O_2_ at 0, 150, and 200 μM for 3 h, washed with PBS, and then incubated with 10 μM DCF-DA (Thermo Fisher Scientific, Wilmington, DE, USA) in serum-free Medium 199 for 30 min at 37 °C. Fluorescence was then measured using a GloMax^®^ Discover Microplate Reader (Promega Corporation, Madison, WI, USA) with excitation at 470 nm and emission at 500–550 nm. Equal numbers of cells were seeded for all experimental groups. ROS levels were expressed as relative DCF fluorescence normalized to the untreated control group.

### 4.6. Immunofluorescence and Myotube Area Analysis

Cells were washed with PBS, fixed in 10% neutral formalin for 15 min, and permeabilized with 0.3% NP-40 for 20 min. Samples were then incubated with PBS containing 1% Tween-20 (PBST) and 5% non-fat dry milk for 30 min. Cells were then incubated with a primary antibody against myosin heavy chain (MyHC; MF20, Developmental Studies Hybridoma Bank, Iowa City, IA, USA) for 1 h. After washing 3 times with PBST for 5 min each, they were incubated with an anti-mouse secondary antibody conjugated to CruzFluor™594 (Santa Cruz Biotechnology) for 1 h. For antibody incubations, the concentration of non-fat dry milk in PBST was reduced to 1%. Nuclei were counterstained with 4′,6-diamidino-2-phenylindole (DAPI) for 5 min. Fluorescence images were acquired using an inverted microscope (CKX53; Olympus, Tokyo, Japan), and the relative area of myotubes was quantified in MyHC-positive cells using Image J software (version 1.54g; National Institutes of Health, Bethesda, MD, USA).

### 4.7. Annexin V/PI Analysis

After a 3 h of treatment with H_2_O_2_, the cells were washed thrice with PBS and incubated with Alexa Fluor 488 Annexin V (Thermo Fisher Scientific) and propidium iodide (PI) according to the manufacturer’s instructions for 30 min at room temperature, in the dark. The cells were then stained with 4′,6-diamidino-2-phenylindole (DAPI), washed with 1X annexin-binding buffer, and imaged using a fluorescence microscope (CKX53; Olympus).

### 4.8. RNA Isolation and Quantitative Real-Time (qRT)-PCR

Total mRNA was extracted using RNAiso Plus (Takara Bio Inc., Shiga, Japan). RNA quality was assessed using electrophoresis, and concentration was measured using on a P200 micro-volume spectrophotometer (Biosis Design, Gwangmyeong-si, Republic of Korea). RNA (1 μg) was converted into cDNA using a DiaStar RT Kit (SolGent, Daejeon, Republic of Korea). RT-qPCR was performed on a CFX Connect™ Real-Time PCR Detection System (Bio-Rad, Hercules, CA, USA) under the following cycling conditions: 95 °C for 3 min, followed by 39 cycles of 95 °C for 15 s, 56–64 °C for 15 s, and 72 °C for 15 s. Relative gene expression was calculated using the 2^−ΔΔCt^ method [47] and *glyceraldehyde-3-phosphate dehydrogenase* (*GAPDH*) as the reference gene (primer sequences are listed in Table 1). 

### 4.9. Western Blotting

Protein was extracted using 1X RIPA buffer supplemented with a protease inhibitor cocktail (GenDEPOT, Baker, TX, USA). Samples were mixed with an equal volume of 2X Laemmli sample buffer (Bio-Rad, Hercules, CA, USA) containing β-mercaptoethanol and boiled at 100 °C for 5 min. Protein concentrations were determined using Coomassie staining. Proteins were separated using SDS-PAGE and transferred onto PVDF membranes. The membranes were blocked with 5% skim milk in 1× Tris-buffered saline containing Tween-20 (TBST) for 1 h. The membranes were incubated with primary antibodies against NRF2 (Bioss Antibodies, Woburn, MA, USA) or α-tubulin at 4 °C overnight. Membranes were then incubated for 1 h with HRP-conjugated goat anti-mouse or anti-rabbit secondary antibodies (Santa Cruz Biotechnology). For incubation with antibodies, the concentration of skim milk in the 1× TBST was lowered to 2.5%. Protein bands were developed using an enhanced chemiluminescence kit (Thermo Fisher Scientific) and imaged using an Amersham ImageQuant 800 (Cytiva, Marlborough, MA, USA).

### 4.10. Statistical Analysis

All data are presented as the mean (±SEM) of at least three independent experiments. Differences between groups were compared using a Student’s *t*-test, one-way ANOVA or two-way ANOVA using an R package (R Foundation for Statistical Computing, Vienna, Austria). Duncan’s multiple range test was applied for post hoc analysis. *p* < 0.05 indicated statistically significant differences.

## Figures and Tables

**Table 1:** List of primers used for cloning and qRT-PCR.

	Primers	Sequence 5′→ 3′	Annealing Temperature	Reference Genes (GenBank No.)
Cloning	STC2-F	5′-CTC gga tcc GAG GAG GAG GAG GGC AAA-3′	64 °C	XM_015876362.2
	STC2 HA-R	5′-CCC tct aga TCA CAG GCT GGC GTA ATC GGG CAC ATC GTA GGG GTA CCT CTG GAT GTC CGA CAA GTC-3′		
qPCR	GAPDH-F	5′-GAG GGT AGT GAA GGC TGC TG-3′	58 °C	XM_015873412.2
	GAPDH-R	5′-ACC AGG AAA CAA GCT TGA CG-3′		
	SOD1-F	5′-ACA CTG CAT CAT TGG CCG TA-3′	58 °C	XM_015881247.1
	SOD1-R	5′-CGA GGT CCA GCA TTT CCA GT-3′		
	SOD2-F	5′-TGC ACT GAA ATT CAA TGG T-3′	56 °C	XM_015858046.1
	SOD2-R	5′-GTT TCT CCT TGA AGT TTG CG-3′		
	GPX1-F	5′-CAA CGG CTT CAA ACC CAA CT-3′	58 °C	AB371709.1
	GPX1-R	5′-GAT GTA CTG CGG GTT GGT CA-3′		
	GPX4-F	5′-GAT CAA AGC CTT TGC GGA GG-3′	58 °C	XM_015886370.2
	GPX4-R	5′-CCA CTT GAT GGC ATT CCC CA-3′		
	HMOX1-F	5′-AGA AGC TGG TGT CCA ACA GC-3′	60 °C	XM_015863488.2
	HMOX1-R	5′-CTG CTA GAC CGT TTT CAT GT-3′		
	CAT-F	5′-TAA GAT GCT GCA GGG TCG TC-3′	64 °C	XM_015863594.1
	CAT-R	5′-ACC TTG GTT GTC AGA CAC ACA-3′		
	GCLC-F	5′-TGT GGA CAC AAG ATG CAC CA-3′	64 °C	XM_015859244.2
	GCLC-R	5′-TGC TTG TAG TCT GGG TGC TG-3′		
	GCLM-F	5′-TCC TTG GAG TAT CTG CAG CC-3′	58 °C	XM_015870026.2
	GCLM-R	5′-TTG GTT TCA CCT GTG CCC AT-3′		
	NQO1-F	5′-GTC GCC GAG CAG AAG AAG AT-3′	60 °C	XM_015874306.1
	NQO1-R	5′-CTT CTT CTG GAA GGG CCC CT-3′		

## Data Availability

Data may be available from the authors upon reasonable request.

## Abbreviations

CAT	Catalase
DAPI	4′,6-diamidino-2-phenylindole
DCF-DA	2′,7′-dichlorofluorescin diacetate
EV	Empty vector
GCLC	Glutamate–cysteine ligase catalytic subunit
GCLM	Glutamate–cysteine ligase modifier subunit
GPX	Glutathione peroxidase
H_2_O_2_	Hydrogen peroxide
HMOX1	Heme oxygenase 1
MyHC	Myosin heavy chain
NQO1	NAD(P)H quinone dehydrogenase 1
NRF2	Nuclear factor erythroid 2-related factor 2
PBS	Phosphate-buffered saline
PI	Propidium iodide
qRT-PCR	Quantitative real-time polymerase chain reaction
ROS	Reactive oxygen species
SOD	Superoxide dismutase
STC2	Stanniocalcin 2
